# Subjective and objective comparisons of image quality between ultra-high-resolution CT and conventional area detector CT in phantoms and cadaveric human lungs

**DOI:** 10.1007/s00330-018-5491-2

**Published:** 2018-05-29

**Authors:** Masahiro Yanagawa, Akinori Hata, Osamu Honda, Noriko Kikuchi, Tomo Miyata, Ayumi Uranishi, Shinsuke Tsukagoshi, Noriyuki Tomiyama

**Affiliations:** 1 Department of Radiology, Osaka University Graduate School of Medicine, 2-2 Yamadaoka, Suita-city, Osaka, 565-0871 Japan; 2 Department of CT Systems, Canon Medical Systems Corp., Otawara, Tochigi Japan

**Keywords:** Multidetector computed tomography, Diagnostic imaging, Lung diseases, Image enhancement, Artifacts

## Abstract

**Objectives:**

To compare the image quality of the lungs between ultra-high-resolution CT (U-HRCT) and conventional area detector CT (AD-CT) images.

**Methods:**

Image data of slit phantoms (0.35, 0.30, and 0.15 mm) and 11 cadaveric human lungs were acquired by both U-HRCT and AD-CT devices. U-HRCT images were obtained with three acquisition modes: normal mode (U-HRCT_*N*_: 896 channels, 0.5 mm × 80 rows; 512 matrix), super-high-resolution mode (U-HRCT_*SHR*_: 1792 channels, 0.25 mm × 160 rows; 1024 matrix), and volume mode (U-HRCT_*SHR-VOL*_: non-helical acquisition with U-HRCT_*SHR*_). AD-CT images were obtained with the same conditions as U-HRCT_*N*_. Three independent observers scored normal anatomical structures (vessels and bronchi), abnormal CT findings (faint nodules, solid nodules, ground-glass opacity, consolidation, emphysema, interlobular septal thickening, intralobular reticular opacities, bronchovascular bundle thickening, bronchiectasis, and honeycombing), noise, artifacts, and overall image quality on a 3-point scale (1 = worst, 2 = equal, 3 = best) compared with U-HRCT_*N*_. Noise values were calculated quantitatively.

**Results:**

U-HRCT could depict a 0.15-mm slit. Both U-HRCT_*SHR*_ and U-HRCT_*SHR-VOL*_ significantly improved visualization of normal anatomical structures and abnormal CT findings, except for intralobular reticular opacities and reduced artifacts, compared with AD-CT (*p* < 0.014). Visually, U-HRCT_*SHR-VOL*_ has less noise than U-HRCT_*SHR*_ and AD-CT (*p* < 0.00001). Quantitative noise values were significantly higher in the following order: U-HRCT_*SHR*_ (mean, 30.41), U-HRCT_*SHR-VOL*_ (26.84), AD-CT (16.03), and U-HRCT_*N*_ (15.14) (*p* < 0.0001). U-HRCT_*SHR*_ and U-HRCT_*SHR-VOL*_ resulted in significantly higher overall image quality than AD-CT and were almost equal to U-HRCT_*N*_ (*p* < 0.0001).

**Conclusions:**

Both U-HRCT_*SHR*_ and U-HRCT_*SHR-VOL*_ can provide higher image quality than AD-CT, while U-HRCT_*SHR-VOL*_ was less noisy than U-HRCT_*SHR*_.

**Key Points:**

*• Ultra-high-resolution CT (U-HRCT) can improve spatial resolution.*

*• U-HRCT can reduce streak and dark band artifacts.*

*• U-HRCT can provide higher image quality than conventional area detector CT.*

*• In U-HRCT, the volume mode is less noisy than the super-high-resolution mode.*

*• U-HRCT may provide more detailed information about the lung anatomy and pathology.*

**Electronic supplementary material:**

The online version of this article (10.1007/s00330-018-5491-2) contains supplementary material, which is available to authorized users.

## Introduction

From the introduction of the first CT device in 1972 to the present day [1], computed tomography (CT) has become an essential imaging modality in a wide range of clinical applications through the incorporation of several innovative technologies. Thin-slice images of the whole lungs can be easily obtained within one breath hold by multidetector-row computed tomography (MDCT) [2]. Both hardware and software have been developed to acquire image data of wide spatial ranges in a short time, facilitated by faster gantry rotation speeds, a widening of detectors [e.g., 320-detector-row CT systems with area detectors (AD-CT)], higher generator power and increased stability of X-ray tubes, and detectors (e.g., garnet-based detectors) available in clinical settings [3–6]. Although the evolution toward faster scanning of a wider range is remarkable, there has been little progress in increasing spatial resolution over the last 30 years.

Regarding the spatial resolution, Imai et al. previously demonstrated improvements in spatial resolution using an experimental MDCT equipped with a density-double matrix detector [1824 channels (x-y plane) × 32 rows (z-axis) at a row width of 0.3125 mm] and an X-ray tube with an ultra-small focal spot [7]. This experimental CT device provided high-resolution imaging while maintaining low-contrast detectability, suggesting a potential for clinical use in areas requiring high spatial resolution, such as imaging of the inner ear, lungs, and bone. In 2017, the ultra-high-resolution CT (U-HRCT) device became available for clinical practice. Kakinuma et al. [8] reported on the performance of a U-HRCT prototype: a 4-row CT device with a detector element size of 0.25 × 0.25 mm at the isocenter and a beam collimation of 0.25 mm × 4 rows. The detector element size of the U-HRCT is half that of a conventional AD-CT in both the in-plane and body-axis directions. Current U-HRCT devices have detectors with 1792 channels in 160 rows. The minimum focus size (0.4 × 0.5 mm) is about a third of the area of a conventional ADCT device (0.9 × 0.8 mm), and the X-ray tube has also improved compared with conventional ADCT devices. By optimizing the relationship between the required radiation dose and focal spot size, a smaller focus size has become operational on the U-HRCT device.

The most advantageous feature of U-HRCT is its improved spatial resolution (120 micron) [8], which makes finer features distinguishable on CT images. No study so far has evaluated the image quality of current U-HRCT acquisitions. The purpose of this study was to compare the image quality of the lungs between U-HRCT and conventional AD-CT.

## Materials and methods

This study was approved by the internal Ethics Review Board of our institute. Informed consent for the retrospective review of patient records and images and use of patient biomaterial was waived.

### Phantom study

Image data of slit phantoms (Kyoto Kagaku Corp., Kyoto, Japan) made of stainless steel were acquired to evaluate the spatial resolution of AD-CT (Aquilion ONE™; Canon Medical Systems Corp., formerly Toshiba Medical Systems, Otawara, Tochigi, Japan) and U-HRCT (Aquilion Precision™; Canon Medical Systems Corp., Otawara, Tochigi, Japan). The slits (0.35, 0.30, and 0.15 mm) and intervening spaces were the same width in each phantom.

Image data of each phantom were acquired with both U-HRCT and AD-CT. The common acquisition parameters were as follows: gantry rotation period, 1.5 s; X-ray voltage, 120 kV_p_; tube current, 200 mA; field of view, 20 mm. The protocol for AD-CT was as follows: the number of channels per detector row, 896 channels and 0.5 mm × 4 rows; matrix size, 512. The U-HRCT was used in super-high-resolution mode (U-HRCT_*SHR*_: the number of channels per detector row, 1792 channels and 0.25 mm × 4 rows; matrix size, 1024). Axial thin-section CT images of 0.5 mm thickness were reconstructed using a lung kernel (FC81): the frequency range of the lung kernel for U-HRCT was twice as wide as that for AD-CT because the number of channels of the U-HRCT device was twice that of the AD-CT device.

### Cadaveric human lungs and imaging

Eleven cadaveric human lungs were inflated and fixed using the Heitzman method [9]. These lungs were distended through the main bronchus with fixative fluid that contained polyethylene glycol 400, 95% ethyl alcohol, 40% formalin, and water in proportions of 10:5:2:3. The specimens were immersed in fixative fluid for 2 days and then air-dried. The pathological diagnoses of these 11 lungs were: pulmonary hemorrhage (*n* = 1), cardiogenic edema (*n* = 1), diffuse panbronchiolitis (*n* = 1), pulmonary tuberculosis (*n* = 2), pulmonary emphysema (*n* = 1), diffuse alveolar damage (*n* = 1), pulmonary metastasis (*n* = 1), pulmonary lymphangitic carcinomatosis (*n* = 1), and usual interstitial pneumonia (*n* = 2).

Image data of the 11 lungs were acquired with both U-HRCT and AD-CT. U-HRCT images were obtained with a 1.5-s gantry rotation, 160 mm field of view, 120 kV_p_, and three types of acquisition modes (see Online Supplementary Material): normal mode [U-HRCT_*N*_: the number of channels per detector row, 896 channels and 0.5 mm × 80 rows; matrix, 512; PF, 0.81; volumetric CT dose index (CTDI_vol_), 23.2 mGy]; super-high-resolution mode (U-HRCT_*SHR*_: the number of channels per detector row, 1792 channels and 0.25 mm × 160 rows; matrix, 1024; PF, 0.81; CTDI_vol_, 23.3 mGy); volume mode (U-HRCT_*SHR-VOL*_: the number of channels per detector row and matrix as in U-HRCT_*SHR*_; CTDI_vol_, 19.2 mGy). AD-CT images were obtained with the same parameters as U-HRCT_*N*_ (but with CTDI_vol_, 23.9 mGy). Image data of the whole lungs were acquired on AD-CT and U-HRCT devices with three acquisition modes, respectively. On U-HRCT_*N*_ images as reference, three cross-sectional levels with the most conspicuous CT findings were selected from each cadaveric human lung by three chest radiologists (A.H., M.Y., and O.H., with 8, 17, and 25 years of experience, respectively) 1 month before starting with the present evaluation. Two technologists (A.U. and S.T.) who were not involved in image evaluations recorded information on the anatomical structures (vessels and bronchi), abnormal CT findings, and artifacts in each cadaveric human lung. We obtained a total of 99 images (33 AD-CT images, 33 U-HRCT_*SHR*_ images, and 33 U-HRCT_*SHR-VOL*_ images) for evaluation and 33 U-HRCT_*N*_ images for reference standard. Both U-HRCT_*SHR*_ images and U-HRCT_*SHR-VOL*_ images had the same three cross-sectional levels as U-HRCT_*N*_ images because all image data were acquired on the same U-HRCT device. AD-CT images had almost the same three cross-sectional levels as U-HRCT_*N*_ images. For comparison with AD-CT images with 0.5 mm thickness, all 132 axial thin-section CT images of 0.5 mm thickness were reconstructed using a lung kernel (FC81) and adaptive iterative dose reduction in three dimensions (ADIR 3D). All CT series were anonymized and transferred to a distant workstation viewer by two technologists (A.U. and S.T.) who were not involved in image evaluation.

### Subjective image interpretation

Three independent chest radiologists (A.H., M.Y., and O.H. with 8, 17, and 25 years of experience, respectively) read all 132 images and evaluated them on a 8.3-megapixel, 32-inch color LCD (4K resolution) monitor without prior knowledge of histopathological diagnoses or image acquisition parameters. For each cadaveric human lung, AD-CT, U-HRCT_*SHR*_, and U-HRCT_*SHR-VOL*_ images were evaluated simultaneously in a blinded manner using U-HRCT_*N*_ images as reference. Images were displayed with a window level of -600 Hounsfield units (HU) and a window width of 1600 HU. The radiologists independently evaluated abnormal CT findings (faint nodules, solid nodules, ground-glass opacity, consolidation, emphysema, interlobular septal thickening, intralobular reticular opacities, bronchovascular bundle thickening, bronchiectasis, and honeycombing), normal anatomical structures (vessels and bronchi), and general aspects of image quality (subjective visual noise, streak artifacts, and dark band artifacts). Overall image quality was also evaluated for each image.

**Fig. 1:**
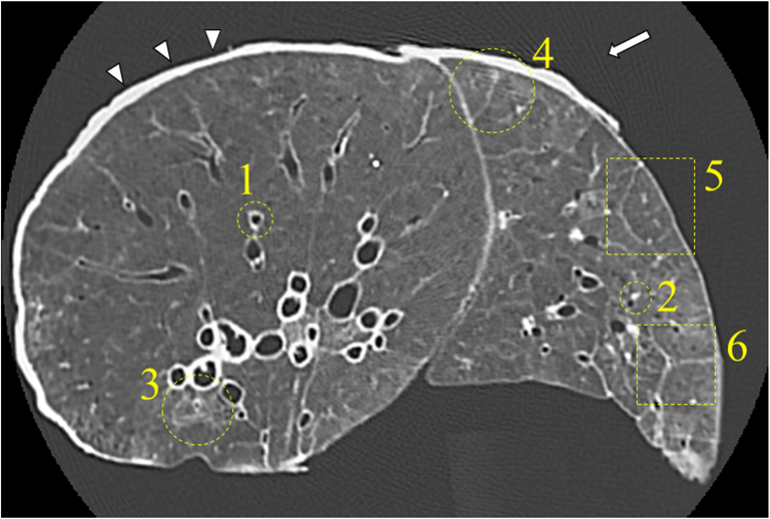
Evaluation items on reference images. On each reference image (U-HRCT_*N*_), each visual evaluation item to be scored is indicated using colored marks. This U-HRCT_*N*_ image of diffuse alveolar damage shows six evaluation items: 1, bronchi; 2, vessels; 3, ground-glass opacity; 4-6, interlobular septal thickening. Streak (arrow) and dark band artifacts (arrowhead) can also be seen. U-HRCT_*N*_: ultra-high-resolution CT with normal mode

Overall image quality, abnormal CT findings, and normal anatomical structures were subjectively graded using a 3-point scale: ‘score 1’ indicated poor image quality (i.e., it was possible to detect structures but difficult to clearly evaluate their margin or internal characteristics); ‘score 2’ indicated fair image quality (i.e., the margin or internal characteristics can be detected and evaluated as well as in the reference images); ‘score 3’ indicated excellent image quality (i.e., it was easy to detect findings and to evaluate their margin or internal characteristics without any indistinct findings). Subjective visual noise and artifacts were also graded on a 3-point scale: ‘score 1’ indicated strong presence; ‘score 2’ indicated moderate presence (i.e., similar to those in the reference images); ‘score 3’ indicated slight presence or almost absence. On each reference image (U-HRCT_*N*_ image), every visual evaluation item to be scored was indicated using colored markers by two technologists (A.U. and S.T.) so that the assessors could identify the position of each visual evaluation item to be evaluated on the other CT images (Fig. 1).

### Objective image interpretation

Quantitative image noise measurements were calculated by measuring the standard deviation (SD) values in circular regions of interest (ROI) drawn on the workstation viewer. Quantitative image noise measurements were obtained from air adjacent to the lungs [10]. ROIs (diameter, 20 mm; area 314 mm^2^) were placed in three homogeneous parts of each image and placed in exactly the same location on each selected image. Average SDs from these three ROIs were computed and compared statistically.

### Statistical analysis

All statistical analyses were performed using commercially available software: MedCalc version 17.6-64-bit statistical software (Frank Schoonjans, Mariakerke, Belgium). Median values of the subjective scores of the three independent radiologists and the statistical significance of any differences among them from the AD-CT, U-HRCT_*SHR*_, and U-HRCT_*SHR-VOL*_ images were assessed using the Friedman test followed by post-hoc tests. Similarly, data from the objective analysis were also analyzed using the Friedman test followed by post-hoc tests. A *p* value < 0.05 was considered significant.

## Results

### Slit phantom evaluation

**Fig. 2:**
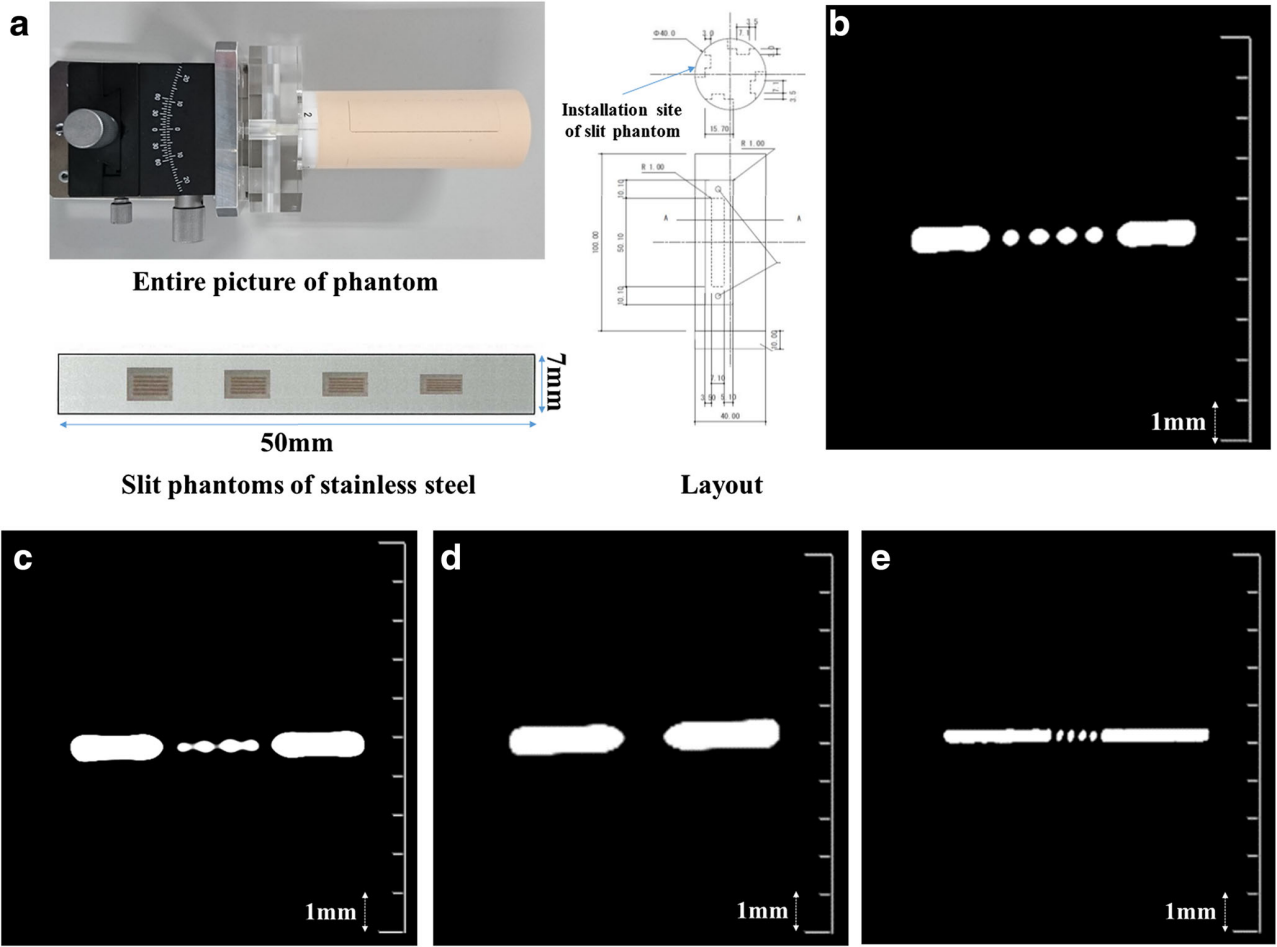
Slit phantom images. Entire picture and layout of the phantom and slit phantoms of stainless steel are shown (**a**). There are four installation sites of slit phantoms. Four stainless steel slit phantoms can be inserted into one installation site at a time (i.e., maximum 16 slit phantoms). In the present study, 0.35-, 0.30-, and 0.15-mm slits were used. AD-CT image with a 20-mm field of view (**b**, **c**, and **d**) and U-HRCT_*SHR*_ image with a 20-mm field of view (**e**). The 0.35-mm slit can be seen in the AD-CT image (**b**) but not clearly with the 0.30-mm slit (**c**). The 0.15-mm slit cannot be seen in the AD-CT image (**d**). However, the 0.15-mm slit can be seen in the U-HRCT_*SHR*_ image (**e**). AD-CT: area detector CT. U-HRCT_*SHR*_: ultra-high-resolution CT with super-high-resolution mode

On AD-CT images, only the 0.35-mm slit could be seen clearly. On U-HRCT_*SHR*_, all the 0.35-mm, 0.30-mm, and 0.15-mm slits could be seen. Therefore, the spatial resolution of AD-CT was at least 0.35 mm and that of U-HRCT_*SHR*_ was at least 0.15 mm (Fig. 2).

### Subjective evaluation: abnormal CT findings

**Fig. 3:**
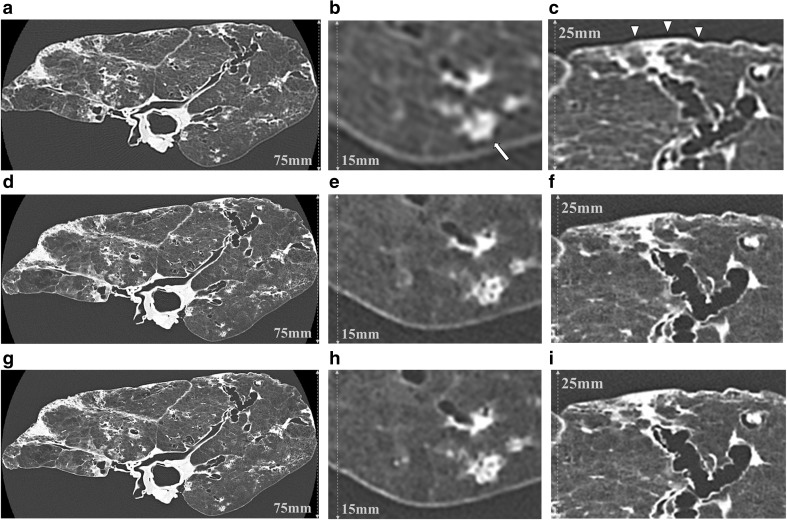
CT images of a cadaveric lung with diffuse panbronchiolitis. Whole and zoomed CT images of AD-CT (**a**, **b**, and **c**), U-HRCT_*SHR*_ (**d**, **e**, and **f**), and U-HRCT_*SHR-VOL*_ (**g**, **h**, and **i**). Tiny (2-mm-diameter) nodules show ill-defined margins and unclear internal structure (**b**). Dark band artifacts (arrowheads) can be seen (**c**). Tiny nodules show well-defined margins and clear internal structure (air bronchiologram) (**e** and **h**). There are almost no dark band artifacts (**f** and **i**). Both U-HRCT_*SHR*_ (**d**) and U-HRCT_*SHR-VOL*_ (**g**) produced significantly better overall image quality than AD-CT (**a**). AD-CT: area detector CT. U-HRCT_*SHR*_: ultra-high-resolution CT with super-high-resolution mode U-HRCT_*SHR-VOL*_: ultra-high-resolution CT with volume mode

**Fig. 4:**
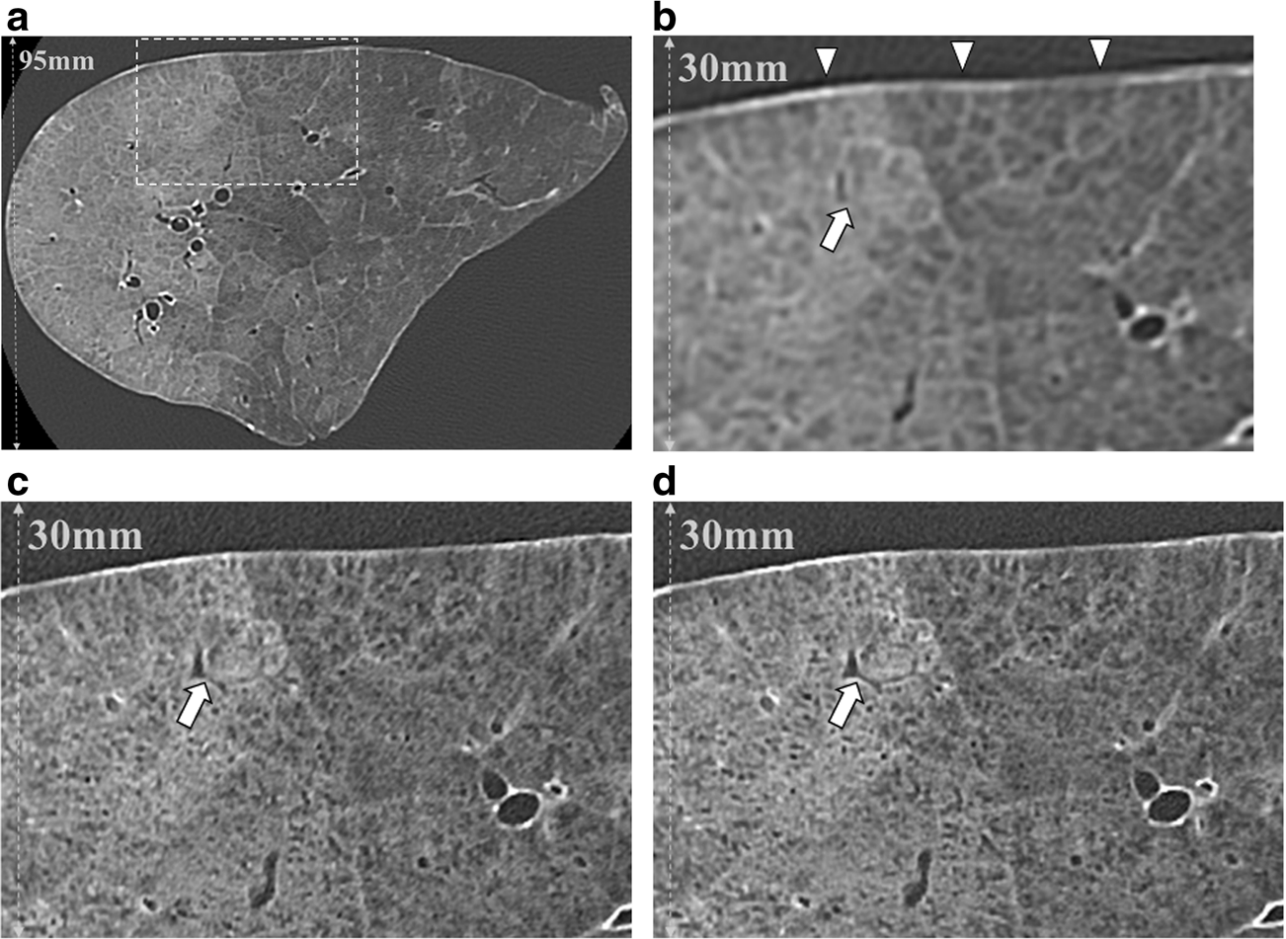
CT images of a cadaveric lung with pulmonary hemorrhage. AD-CT (area detector CT) image of a cadaveric lung with pulmonary hemorrhage (**a**). Zoomed CT images corresponding to a dashed rectangle in (**a**) are shown (**b**, **c**, and **d**). Intralobular reticular opacities can be detected more easily on AD-CT (**b**) than on U-HRCT_*SHR*_ (**c**) and U-HRCT_*SHR-VOL*_ (**d**). On the other hand, normal anatomical structures such as bronchi and vessels (arrow) can be seen more clearly on U-HRCT_*SHR*_ (**c**) and U-HRCT_*SHR-VOL*_ (**d**) than on AD-CT (**b**). U-HRCT_*SHR*_ (**c**) and U-HRCT_*SHR-VOL*_ (**d**) seem to also show normal anatomical structures such as small bronchi and vessels as low attenuation areas. While dark band artifacts (arrowheads) can be seen in (**b**), there are almost no dark band artifacts in (**c** and **d**).

The image quality scores for abnormal CT findings on AD-CT, U-HRCT_*SHR*_, and U-HRCT_*SHR-VOL*_ are summarized in Table 1. Both U-HRCT_*SHR*_ and U-HRCT_*SHR-VOL*_ abnormal CT finding scores were significantly higher than those of AD-CT (*p* < 0.0001) (Fig. 3) except for intralobular reticular opacities. For intralobular reticular opacities, AD-CT abnormal CT finding scores were significantly higher than those of U-HRCT_*SHR*_ and U-HRCT_*SHR-VOL*_ (*p* < 0.0014) (Fig. 4).

**Table 1:** Subjective evaluation: abnormal CT findings

	Abnormal CT Findings									
	Faint nodules *N* = 11	Solid nodules *N* = 26	GGO *N* = 25	Consolidation *N* = 11	Emphysema *N* = 12	ISP *N* = 15	IRO *N* = 12	BBT *N* = 12	Bronchiectasis *N* = 9	Honeycombing *N* = 11
Acquisition mode										
AD-CT	1.72 ± 0.46*¶	1.92 ± 0.27*¶	1.92 ± 0.27*¶	1.90 ± 0.30*¶	2.00 ± 0.00*¶	2.00 ± 0.00*¶	2.08 ± 0.28*¶	2.00 ± 0.00*¶	2.00 ± 0.00*¶	2.00 ± 0.00*¶
U-HRCT_*SHR*_	3.00 ± 0.00*	3.00 ± 0.00*	2.92 ± 0.27*	3.00 ± 0.00*	3.00 ± 0.00*	3.00 ± 0.00*	1.58 ± 0.66*	3.00 ± 0.00*	3.00 ± 0.00*	3.00 ± 0.00*
U-HRCT_*SHR-VOL*_	3.00 ± 0.00¶	3.00 ± 0.00¶	2.92 ± 0.27¶	3.00 ± 0.00¶	3.00 ± 0.00¶	3.00 ± 0.00¶	1.66 ± 0.65¶	3.00 ± 0.00¶	3.00 ± 0.00¶	3.00 ± 0.00¶

Data are presented as mean ± SD. Data of the subjective image analysis were statistically analyzed using the Friedman test followed by post-hoc tests.

GGO = ground-glass opacity, ISP = interlobular septal thickening, IRO = intralobular reticular opacities, BBT = bronchovascular bundle thickening.

AD-CT: area detector CT

U-HRCT_*SHR*_: ultra-high-resolution CT with super-high-resolution mode.

U-HRCT_*SHR-VOL*_: ultra-high-resolution CT with volume mode.

*There was a significant difference between AD-CT and U-HRCT_*SHR*_ (*p* < 0.05).

¶There was a significant difference between AD-CT and U-HRCT_*SHR-VOL*_ (*p* < 0.05)

### Subjective evaluation: normal anatomical structures and general aspects of image quality

The image quality scores for normal anatomical structures and general aspects of image quality on AD-CT, U-HRCT_*SHR*_, and U-HRCT_*SHR-VOL*_ are summarized in Table 2. Both U-HRCT_*SHR*_ and U-HRCT_*SHR-VOL*_ normal anatomical structure scores for bronchi and vessels were significantly higher than those of AD-CT (*p* < 0.0001) (Fig. 4). Regarding the general aspects of image quality, both U-HCT_*SHR*_ and U-HRCT_*SHR-VOL*_ indicated significantly decreased streak and dark band artifacts with respect to AD-CT (*p* < 0.0001) (Figs. 3 and 4). The subjective visual noise of U-HRCT_*SHR-VOL*_ was the lowest of the three groups (*p* < 0.00001). U-HRCT_*SHR*_ and U-HRCT_*SHR-VOL*_ overall image quality scores were significantly higher than those of AD-CT and almost equal to those of U-HRCT_*N*_ (*p* < 0.0001).

**Table 2:** Subjective evaluation: normal anatomical structures and general aspects of image quality

	Normal anatomical structures		General aspects of image quality			Overall image quality
	Bronchi *N* = 29	Vessels *N* = 29	Subjective visual noise *N* = 33	Streak artifacts *N* = 33	Dark band artifacts *N* = 33	*N* = 33
Acquisition mode						
AD-CT	1.93 ± 0.25*¶	1.86 ± 0.35*¶	2.00 ± 0.00¶	2.30 ± 0.68*¶	2.00 ± 0.00*¶	2.00 ± 0.00*¶
U-HRCT_*SHR*_	3.00 ± 0.00*	3.00 ± 0.00*	2.00 ± 0.35#	2.93 ± 0.34*	3.00 ± 0.00*	3.00 ± 0.00*
U-HRCT_*SHR-VOL*_	3.00 ± 0.00¶	3.00 ± 0.00¶	2.39 ± 0.49¶#	3.00 ± 0.00¶	3.00 ± 0.00¶	3.00 ± 0.00¶

Data are presented as mean ± SD. Data of the subjective image analysis were statistically analyzed using the Friedman test followed by post-hoc tests.

AD-CT: area detector CT.

U-HRCT_*SHR*_: ultra-high-resolution CT with super-high-resolution mode.

U-HRCT_*SHR-VOL*_: ultra-high-resolution CT with volume mode.

*There was a significant difference between AD-CT and U-HRCT_*SHR*_ (*p* < 0.0001)

¶There was a significant difference between AD-CT and U-HRCT _*SHR-VOL*_ (*p* < 0.0001)

#There was a significant difference between U-HRCT_*SHR*_ and U-HRCT _*SHR-VOL*_ (*p* < 0.0001)

### Quantitative image noise measurements

Quantitative noise values (mean ± SD) on CT images were as follows: AD-CT (16.03 ± 5.17), U-HRCT_*N*_ (15.14 ± 5.49), U-HRCT_*SHR*_ (30.41 ± 4.65), and U-HRCT_*SHR-VOL*_ (26.84 ± 5.12). Quantitative noise values were significantly higher in the following order: U-HRCT_*SHR*_, U-HRCT_*SHR-VOL*_, AD-CT, and U-HRCT_*N*_ (*p* < 0.0001).

## Discussion

This study showed that ultra-high-resolution CT (U-HRCT_*SHR*_ and U-HRCT_*SHR-VOL*_) significantly improved the visualization of normal and abnormal CT findings compared with AD-CT, except for intralobular reticular opacities and reduced streak and dark band artifacts. U-HRCT_*SHR*_ and U-HRCT_*SHR-VOL*_ provided significantly higher overall image quality than AD-CT. In particular, U-HRCT_*SHR-VOL*_ was less noisy than U-HRCT_*SHR*_. The present study is the first to evaluate the image quality of U-HRCT images compared with AD-CT. The use of U-HRCT might enhance image quality by improving spatial resolution, resulting in provision of more detailed information of lung anatomy and pathology. However, our results might be speculative from a clinical point of view, as all data in the present study originated from an ex-vivo phantom study employing explanted lungs imaged free in air. Further analyses are needed to validate our results by using larger cohorts including various diseases in a clinical practice.

The recent development of U-HRCT technology (i.e., 0.25 × 0.25 mm detector element size, a detector with 1792 channels in 160 rows, and 0.4 × 0.5 mm minimum focus size of the X-ray tube) improve spatial resolution in both in-plane and body-axis directions. In the present evaluation of a slit phantom using a reconstruction FOV of 20 mm, the spatial resolution of U-HRCT (at least 0.15 mm) was at least two times higher than that of AD-CT (at least 0.35 mm). In the evaluation of cadaveric human lungs using a reconstruction FOV of 160 mm, the pixel sizes in 512 × 512 (AD-CT) and 1024 × 1024 matrices (U-HRCT_*SHR*_ and U-HRCT_*SHR-VOL*_) were 0.313 mm and 0.156 mm, respectively. In this study, ADCT could resolve up to 0.35 mm in spatial resolution (0.313-mm pixel size < 0.35-mm maximum spatial resolution of AD-CT). On the other hand, U-HRCT could resolve up to 0.156 mm in spatial resolution (0.156-mm pixel size > 0.15-mm maximum spatial resolution of U-HRCT). Therefore, it is important to understand the limit of spatial resolution in each CT device and confirm the optimal conditions to secure the spatial resolution in a clinical setting.

Although spatial resolution depends on the matrix size of the reconstructed images, it can never be higher than the maximum spatial resolution of the CT device itself. U-HRCT makes it possible to reconstruct images with matrixes larger than 512 × 512, which is common nowadays [11]. By using a larger matrix and reducing the pixel size, U-HRCT can provide higher spatial resolution for the same FOV size. However, it is important to select a matrix size that is suitable for the intrinsic resolution of the device, determined by the focus size and the detector element size. Further studies of U-HRCT are needed to examine the effect of the matrix size on image quality.

In our evaluation of abnormal CT findings in cadaveric human lungs, both U-HRCT_*SHR*_ and U-HRCT_*SHR-VOL*_ significantly improved the abnormal CT findings (ground-glass opacity, consolidation, emphysema, faint and solid nodules, interlobular septal thickening, bronchiectasis, honeycombing) compared with AD-CT. A possible reason why these abnormal findings were more conspicuous in U-HRCT could be the increased spatial resolution of the U-HRCT device. Regarding intralobular reticular opacities, however, visual score values for AD-CT were significantly higher than for U-HRCT_*SHR*_ and U-HRCT_*SHR-VOL*_. In general, intralobular reticular opacities refer to the appearance on HRCT of scattered or diffuse ground-glass attenuation with superimposed interlobular septal thickening and intralobular lines [12–14]. This CT finding is due to interstitial pulmonary abnormalities and/or alveolar abnormalities [12, 15–17]. We speculate some of the reasons could be the superior spatial resolution of U-HRCT enables visualizing the fine structures around the interlobular septum, resulting in the relative blurring of linear shadows in a case of images including two kinds of abnormal CT findings such as intralobular reticular opacities. In other words, fine shadows are recognized as ground-glass attenuation because of a partial volume effect on AD-CT due to an inferior spatial resolution. As a result, interlobular septal thickening might be relatively conspicuous compared with the surrounding ground-glass attenuation. Even in those cases that showed the same CT findings as on AD-CT, the higher spatial resolution of U-HRCT might provide further information on the origin of these CT findings. In the future, it will be necessary to correlate the CT findings in various diseases with pathological specimens.

In our evaluation of normal anatomical structures and artifacts in cadaveric human lungs, both U-HRCT_*SHR*_ and U-HRCT_*SHR-VOL*_ significantly improved the visualization of normal anatomical structures such as bronchi and vessels and reduced streak and dark band artifacts compared with AD-CT. In the case of abnormal CT findings, the higher spatial resolution produced by the U-HRCT device can help better visualize thin linear opacities such as bronchi and vessels. U-HRCT might be able to reduce streak artifacts because the 1792 channels of the detector element affect the resolution of the X-Y plane enabling more precise sampling [18]. Moreover, the frequency range of the reconstruction algorithm is doubled in U-HRCT as the number of channels is twice that of AD-CT. Therefore, it is not necessary to forcibly emphasize the high spatial frequency region as is done in AD-CT, resulting in almost no dark band artifacts caused by an undershoot.

Regarding image noise, quantitative noise values were significantly higher in the following order: U-HRCT_*SHR*_, U-HRCT_*SHR-VOL*_, and AD-CT. The subjective visual noise of U-HRCT_*SHR-VOL*_, however, was the lowest. This might be due to the visual effects associated with the overall higher image quality of U-HRCT_*SHR-VOL*_. In this study, AIDR3D, a hybrid iterative reconstruction method, was used for reducing image noise. Previous papers have shown that model-based iterative reconstruction imaging could provide higher image quality with lower noise and artifacts [19, 20]. In the future, the combined use of model-based iterative reconstruction and U-HRCT could possibly result in even higher image quality. As a whole, U-HRCT_*SHR*_ and U-HRCT_*SHR-VOL*_ produced a higher overall image quality than AD-CT, almost equal to U-HRCT_*N*_*,* by precisely delineating fine and/or thin structures and reducing artifacts. U-HRCT_*SHR-VOL*_ is less noisy than U-HRCT_*SHR*_. Regarding the improvements of spatial resolution, Fischbach et al. [21] demonstrated that thin section images enhanced resolution, decreased volume averaging from slice to slice, and resulted in an improvement of small nodule detection, confidence levels, and interobserver agreement. Coche et al. [22] demonstrated that enhanced multislice spiral CT with thin collimation could be used to analyze the subsegmental pulmonary arteries precisely and might identify even more distal pulmonary arteries. Yoshioka et al. [23] demonstrated that U-HRCT with 0.25-mm slices significantly improved the visualization of the artery of Adamkiewicz compared with 0.5-mm slices. Therefore, in the future, the improvement of spatial resolution on U-HRCT might have the possibility to lead to diagnostic imaging advances in the lungs. Simultaneously, it might be also important to investigate how detailed CT findings will affect the patient's outcome.

There are several limitations to the study. First, this study included only a small number of cases with a few limited CT findings. Second, evaluations of the influence of absorption and scattering in the human thorax on image quality were lacking. No influence of motion artifacts on image quality was evaluated because of the use of cadaveric human lungs. Therefore, in the future, it will be necessary to investigate how these factors influence the image quality of U-HRCT. Third, image quality was evaluated using CT devices manufactured by a single company. Presently, however, there are no CT devices that offer a similar performance as the one used in this study. In the future, we expect that U-HRCT devices developed by other companies will be clinically available. Fourth, regarding the slice position of CT images, we made the utmost effort to get the same cross section images from the different CT devices. However, we could not get exactly the same images. Fifth, the maximum matrix size in this study was 1024, and further analyses are needed to assess the effect of various matrix sizes on the image quality of U-HRCT. Sixth, there was a potential source of error concerning the radiation exposure. Radiation exposure of the volume mode in terms of CTDI_vol_ was about 15% less than the other acquisition modes. Although the maximum effort was made so that the CTDI_vol_ values would be almost equivalent among the acquisition settings, the tube current could only be adjusted by 10-mA increments on each CT device (i.e., AD-CT, U-HRCT). Ideally, image noise should be evaluated with exactly the same radiation exposure. Moreover, the radiation dose with CTDI_vol_ higher than 20 mGy used in cadaveric human lungs without chest walls might be high and could not be applied with a similar size-specific dose estimation to humans. In a practical setting, evaluations of lung image quality on U-HRCT is needed under the appropriate radiation dose. Seventh, although we evaluated CT findings in pathologically diagnosed cadaveric human lungs, no detailed correlations of CT findings with pathological specimens were assessed. Pathological correlation would be needed to investigate small-size CT findings, including intralobular reticular opacities, in the future. Finally, regarding the 11 cadaveric human lungs, they were specimens that had been stored in our institution for a long time. Although the 11 lungs were inflated and fixed by the Heitzman method and were diagnosed by a pathologist, the details (i.e., type of imaging technique, acquisition, and reading procedure) were unknown. We could not consider the influence of the specimen fixation method and preservation condition on the pathological diagnosis. At least, however, the conditions of cadaveric human lungs at the time of imaging were the same because their image data were acquired on both AD-CT and U-HRCT devices around almost the same time.

In conclusion, U-HRCT (U-HRCT_*SHR*_ and U-HRCT_*SHR-VOL*_) can provide higher image quality than AD-CT by improving the spatial resolution and reducing artifacts. U-HRCT_*SHR-VOL*_ is also more advantageous concerning noise than U-HRCT_*SHR*_. U-HRCT may provide more detailed information for lung anatomy and pathology by clearly delineating CT findings (e.g., vessels, bronchi, ground-glass opacity, consolidation, emphysema, faint and solid nodules, interlobular septal thickening, bronchiectasis, and honeycombing).

## Electronic supplementary material

ESM 1 (PNG 16.6 kb)

High resolution image (TIF 2049 kb)

## Abbreviations

AD-CT: Conventional area detector computed tomography
ADIR 3D: Adaptive iterative dose reduction in three dimensions
CT: Computed tomography
CTDIvol: Volumetric computed tomography dose index
HU: Hounsfield units
MDCT: Multidetector row computed tomography
U-HRCT: Ultra-high-resolution computed tomography
U-HRCT_N_: Ultra-high-resolution computed tomography with normal mode
U-HRCT_SHR-VOL_: Ultra-high-resolution computed tomography with volume mode
U-HRCT_SHR_: Ultra-high-resolution computed tomography with super-high-resolution mode
