# Supplementary figures and images for: Subjective and objective comparisons of image quality between ultra-high-resolution CT and conventional area detector CT in phantoms and cadaveric human lungs
Source: Eur Radiol. 2018 May 29;28(12):5060–8. doi: 10.1007/s00330-018-5491-2 (PMC6223853; doi:10.1007/s00330-018-5491-2)

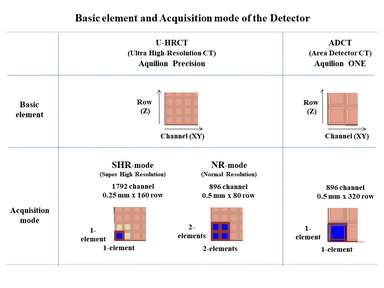

Supplement: Supplementary file 1 — (PNG 16.6 kb) [file 330_2018_5491_Fig5_ESM.png]

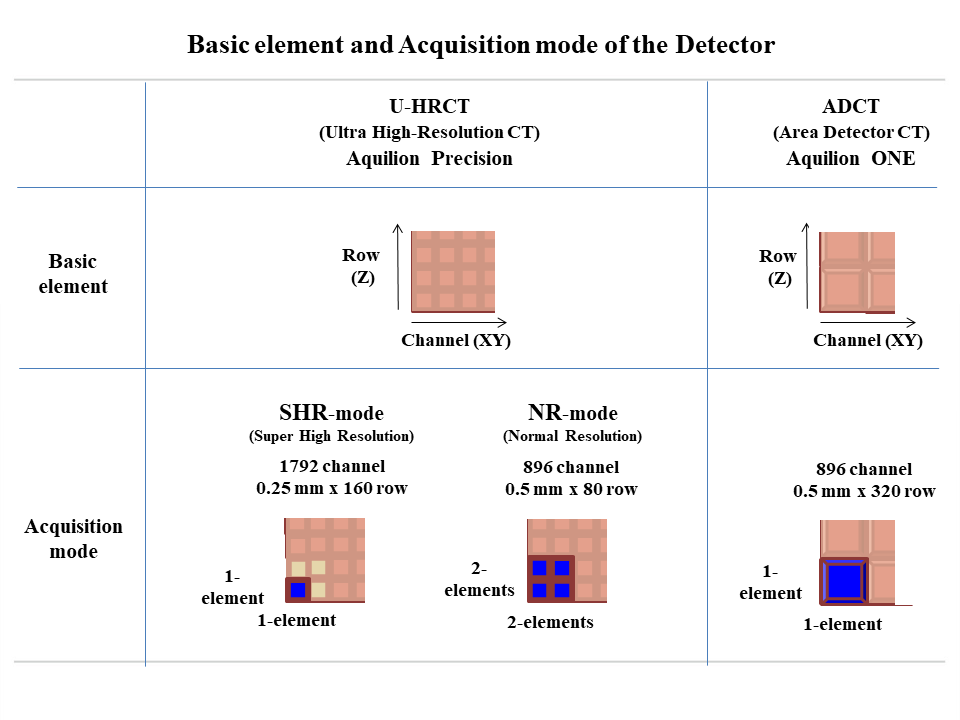

Supplement: Supplementary file 2 — High resolution image (TIF 2049 kb) [file 330_2018_5491_MOESM1_ESM.tif]
